# ﻿A review of the *Temnothoraxanodontoides* species-group (Hymenoptera, Formicidae) from Greece

**DOI:** 10.3897/zookeys.1091.79085

**Published:** 2022-03-01

**Authors:** Sebastian Salata, Lech Borowiec

**Affiliations:** 1 University of Wrocław, Department of Biodiversity and Evolutionary Taxonomy, Myrmecological Laboratory, Przybyszewskiego 65, 51-148 Wrocław, Poland University of Wrocław Wrocław Poland

**Keywords:** Myrmicinae, new species, *Temnothoraxanodontoides* group, taxonomy

## Abstract

A review of the Greek members of the *Temnothoraxanodontoides* species-group revealed three species new to science: *Temnothoraxeuboeae***sp. nov.** (Sterea Ellas, Euboea Island), *Temnothoraxarkasi***sp. nov.** (Peloponnese, Arcadia) and *Temnothoraxparnonensis***sp. nov.** (Peloponnese, Arcadia and Lakonia). The diagnoses of *Temnothoraxikarosi* Salata, Borowiec & Trichas, 2018 and *T.anodontoides* (Dlussky & Zabelin, 1985) are updated based of the new discoveries. Additionally, presence of the latter species in Greece is discussed and its distribution range revised. All members of the *anodontoides* species-group are associated with alpine and rocky habitats such as pastures and thermophilous forests. A dichotomous key to the *anodontoides* species-group from Greece is given.

## ﻿Introduction

The myrmicine genus *Temnothorax* Mayr, 1861, with 452 valid species and 36 valid subspecies, is one of the most speciose ant genera (Bolton 2021). Most of its members are distributed in the Northern Hemisphere, with diversity centers located in the Mediterranean region, southern parts of the USA, and the Greater Antilles (Salata and Borowiec 2019; Bolton 2021; Prebus 2021). *Temnothorax* species occupy a wide range of habitats, including tropical rainforests, hot deserts and boreal forests. Members of this genus nest most often in small, preformed cavities, such as rock crevices, hollow dead twigs, and dry acorns. They nest also under moss on stones and directly in ground, occasionally under cracked bark on tree trunks (Prebus 2021). Among 285 Palaearctic taxa, which consist of 59% of all known *Temnothorax* species, nearly 200, i.e., ~40% of all *Temnothorax*, are known from the Mediterranean region (sensu Vigna Taglianti et al. 1999). Due to its diversity, the Mediterranean myrmecofauna has been recently a subject of thorough studies that were partially focused on this genus (Csősz et al. 2015, 2018; Radchenko et al. 2015; Salata and Borowiec 2015; Galkowski and Lebas 2016; Catarineu et al. 2017; Galkowski and Cagniant 2017; Sharaf et al. 2017; Salata et al. 2018; Salata and Borowiec 2019; Tinaut and Reyes–López 2020; Arcos González 2021), and resulted in the description of several species new to science.

The *Temnothoraxanodontoides* species-group was for the very first time defined by Salata and Borowiec (2019) and referred to the Balkan species characterized by the following set of characters: 12-segmented antennae, darkened club, absence of metanotal groove, overall body coloration brown to almost black, propodeal spines absent or short with wide base, rounded or at most subangulate petiolar node in profile, and very strongly sculptured head and mesosomal surface. Overall, the morphological definition of the *anodontoides* species-group partly overlaps with the *korbi* species-group defined by Radchenko (1995). However, *T.anodontoides* was excluded from the *korbi* species-group because it was the only member with distinct head sculpture, dark body colouration and unique shape of the petiole. The remaining members of the *korbi* group, i.e., *T.korbi* (Emery, 1924), *T.caucasicus* (Arnoldi, 1977) (now junior synonym of *T.nadigi*), *T.anodonta* (Arnoldi, 1977), and *T.iranicus* (Radchenko, 1994), form a separate group more closely related to the *bulgaricus* group (sensu Salata and Borowiec 2019). In this new sense the *anodontoides* species-group covers species recorded from the Apennines, Balkans and Kopet Dag Mts at the border area between Iran and Turkmenistan. Based on the literature, the Balkans hosts only two species of the *anodontoides* group: *Temnothoraxanodontoides* (Dlussky & Zabelin, 1985), described from Turkmenistan close to the Iranian border, and *Temnothoraxikarosi* Salata, Borowiec & Trichas, 2018, described from Crete. *Temnothoraxanodontoides* was noted from subalpine meadows and its nests were located directly in the ground (Dlussky and Zabelin 1985). Whereas *T.ikarosi* was described recently from the Limnakarou Plateau on Crete. A single specimen of this species was collected on a shelter wall overgrown by blackberry bush (Salata et al. 2018).

Occurrence of members of the *anodontoides* species-group in Greece was for the first time suggested by Schulz et al. (2007). In the paper on Italian *Temnothorax*, the authors compared therein described *Temnothoraxsaxatilis* Schulz, Heinze & Pusch, 2007 with a Greek taxon collected in Arcadia, Peloponnese that was tentatively identified as *Temnothoraxanodontoides*. By courtesy of Alex Radchenko (UASK, Kiev), Petr Werner (Prague), and Claude Lebas (Canohès), we had an opportunity to study a paratype specimen of *T.anodontoides*, a series of specimens collected in Arcadia and identified by A. Schulz as *T.anodontoides*, and other material collected from Greek mountains with characters of the *anodontoides* species-group. Based on our research, we concluded that there are four Balkan species belonging to the *anodontoides* species-group: *T.ikarosi* known from Crete (Salata et al. 2018), two species known from the Greek mainland: *Temnothoraxarkasi* sp. nov. and *Temnothoraxparnonensis* sp. nov., and *Temnothoraxeuboeae* sp. nov., so far known only from Euboea Island. The literature records of *T.anodontoides* from Greece (Schulz et al. 2007) should be assigned to *T.arkasi* sp. nov. and its presence in Europe is doubtful. Also, we consider *T.saxatilis* as a member of the *anodontoides* species-group but due to its absence in Greece we did not include it in the review. However, when necessary, we included this species in the differential diagnoses of the species described as new to science. Below, we describe three species new to science, provide their photographs and a key to all members of the *anodontoides* species-group known from Greece.

## ﻿Materials and methods

### ﻿Examined specimens are housed in the following collections

**MHNG**Museum d’Historie Naturelle, Geneve, Switzerland;

**MNHW-DBET** Museum of Natural History, University of Wrocław, in temporary deposit by Department of Biodiversity and Evolutionary Taxonomy, University of Wrocław, Poland;

**PWC** private collection of Petr Werner, Prague, Czech Republic;

**UASK**Institute of Zoology, National Academy of Sciences of Ukraine, Kiev.

Specimens were compared using standard methods of comparative morphology. All measurements were made in μm using a pin-holding stage, permitting rotations around X, Y, and Z axes. A Nikon SMZ18 stereomicroscope was used at a magnification of ×100 for each character. Photographs were taken using a Nikon SMZ 1500 stereomicroscope, Nikon D5200 camera and Helicon Focus software. All given label data of type specimens are in original spelling, presented in square brackets; a vertical bar (|) separates data on different rows and double vertical bars (||) separate labels. Images of type specimens are available online on AntWeb (www.AntWeb.org) and are accessible using the unique identifying specimen codes provided in the description sections.

Pilosity inclination measurements follow Wilson (1955): adpressed (0–5°) hairs run parallel, or nearly parallel to the body surface; decumbent hairs stand 10–15°; subdecumbent hair stands 30°; suberect hairs stand 35–45°; and erect hairs stand more than 45° from the body surface. The surface sculpturing glossary follows Harris (1979).

### ﻿Measurements

**EL** eye length; measured along the maximum diameter of the eye;

**EW** eye width; measured along the minimum diameter of the eye;

**HL** head length; measured in straight line from mid-point of anterior clypeal margin to mid-point of posterior margin in full-face view (i.e., when both maximum head length in median line and maximum head width are positioned in visual plane);

**HW** head width; measured in full-face view directly posterior of the eyes;

**PEH** petiole height; measured in lateral view, the chord of ventral petiolar profile at node level is the reference line perpendicular to which the maximum height of petiole is measured (fig. 1D in Csősz et al. 2015);

**PEL** petiole length; measured in lateral view, from anterior corner of subpetiolar process to dorsocaudal corner of caudal cylinder (fig. 3 in Csősz and Fisher 2015);

**PNW** pronotum width; maximum width of pronotum in dorsal view;

**PPH** postpetiole height; measured perpendicularly to a line defined by the linear section of the segment border between dorsal and ventral petiolar sclerite (fig. 1D in Csősz et al. 2015);

**PPL** postpetiole length; maximum length of the postpetiole measured in lateral view perpendicular to the straight section of lateral postpetiolar margin (fig. 1D in Csősz et al. 2015);

**PPW** postpetiole width; maximum width of postpetiole in dorsal view;

**PSL** propodeal spine length; measured from the centre of the propodeal spiracle to the top of the propodeal spine;

**PEW** petiole width; maximum width of petiole in dorsal view;

**SDL** spiracle to declivity length; minimum distance from the centre of the propodeal spiracle to the propodeal declivity;

**SL** scape length; maximum straight-line length of scape excluding the articular condyle;

**WL** mesosoma length; measured as diagonal length from the anterior end of the neck shield to the posterior margin of the propodeal lobe.

### ﻿Indices

**CI**HW/HL;

**EI1**EW/EL;

**EI2**EW/HL;

**SI1**SL/HL;

**SI2**SL/HW;

**MI**PNW/WL;

**PI**PEL/PEH;

**PPI**PPL/PPH;

**PSI**PSL/SDL.

### ﻿Abbreviations

**w.** worker

## ﻿Results

### ﻿Synopsis of the *Temnothoraxanodonotoides* species-group known from Greece

*Temnothoraxarkasi* sp. nov.

*Temnothoraxeuboeae* sp. nov.

*Temnothoraxikarosi* Salata, Borowiec & Trichas, 2018

*Temnothoraxparnonensis* sp. nov.

### ﻿Key to members of the *Temnothoraxanodonotoides* species-group known from Greece

**Table:** 

1	Head with reduced sculpture, frons medially with long and narrow smooth or indistinctly punctate area (Fig. 15). Body predominantly yellowish brown (Figs 9, 10). Mountains of Peloponnese	***T.parnonensis* sp. nov.**
–	Head strongly sculptured, distinctly reticulate (Figs 7, 8). Body predominantly brown to almost black (Figs 1, 2, 5, 6, 17, 18)	**2**
2	Petiole with short peduncle (Fig. 6). Propodeal spines well marked, needle shaped (Fig. 6). Euboea	***T.euboeae* sp. nov.**
–	Petiole with elongated peduncle (Figs 2, 18). Propodeal spines absent or short, in form of triangular denticle (Figs 2, 18)	**3**
3	Petiole regularly rounded in profile, with shorter peduncle (Fig. 2), petiolar and postpetiolar dorsum with distinct irregular rugae; promesonotum with denser and thinner sculpture (Fig. 1). Peloponnese	***Temnothoraxarkasi* sp. nov. (= *T.anodontoides* sensu Schulz et al. 2007)**
–	Petiole subangulate in profile, with longer peduncle (Fig. 18), petiolar and postpetiolar dorsum reticulate, rugae absent, promesonotum with thicker and sparser sculpture (Fig. 17). Crete	***T.ikarosi* Salata, Borowiec & Trichas**

### ﻿Species accounts

**Note.** Because of the partly reduced head sculpture, *Temnothoraxparnonensis* doesn’t entirely match the characteristics of the *anodontoides* species-group proposed by Salata and Borowiec (2019). However, because its habitat preferences and overall morphology match most of the characters associated with this group, we decided to include it in the revision. Based on that, the definition of the *anodontoides* species-group should be modified as follow: 12-segmented antennae, darkened club, absence of metanotal groove, overall body coloration from yellowish brown to almost black, propodeal spines absent or short with wide base, rounded or at most subangulate petiolar node in profile, very strongly sculptured mesosomal surface; head strongly sculptured or strongly sculptured with frons with diffused sculpture and sometimes medially with narrow smooth area.

#### ﻿ Temnothorax arkasi sp. nov.

Taxon classificationAnimaliaHymenopteraFormicidae

36718DDF-3718-5ADB-9CDB-900A29AC4CC7

http://zoobank.org/23D7FD80-558A-47D9-832C-B797F46B4A6B

**Figures 1, 2.**
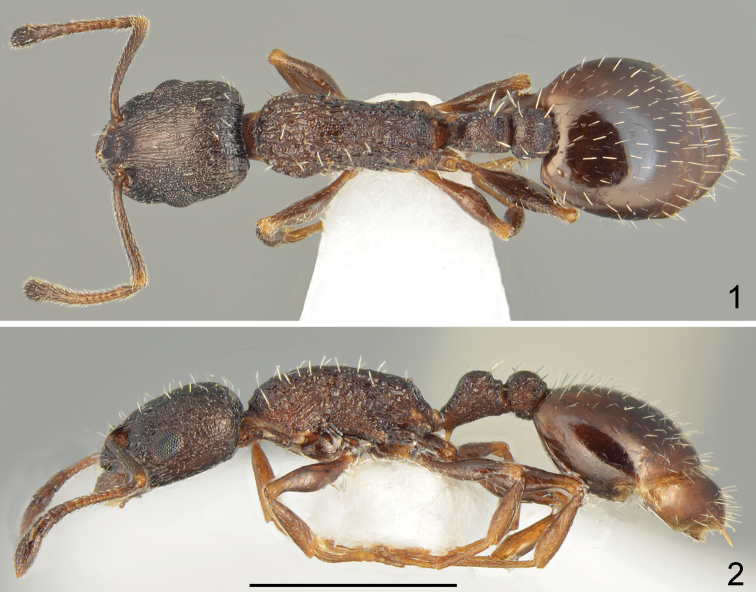
Holotype worker of *Temnothoraxarkasi* sp. nov. **1** dorsal **2** lateral. Scale bar: 1 mm.

**Figures 3, 4.**
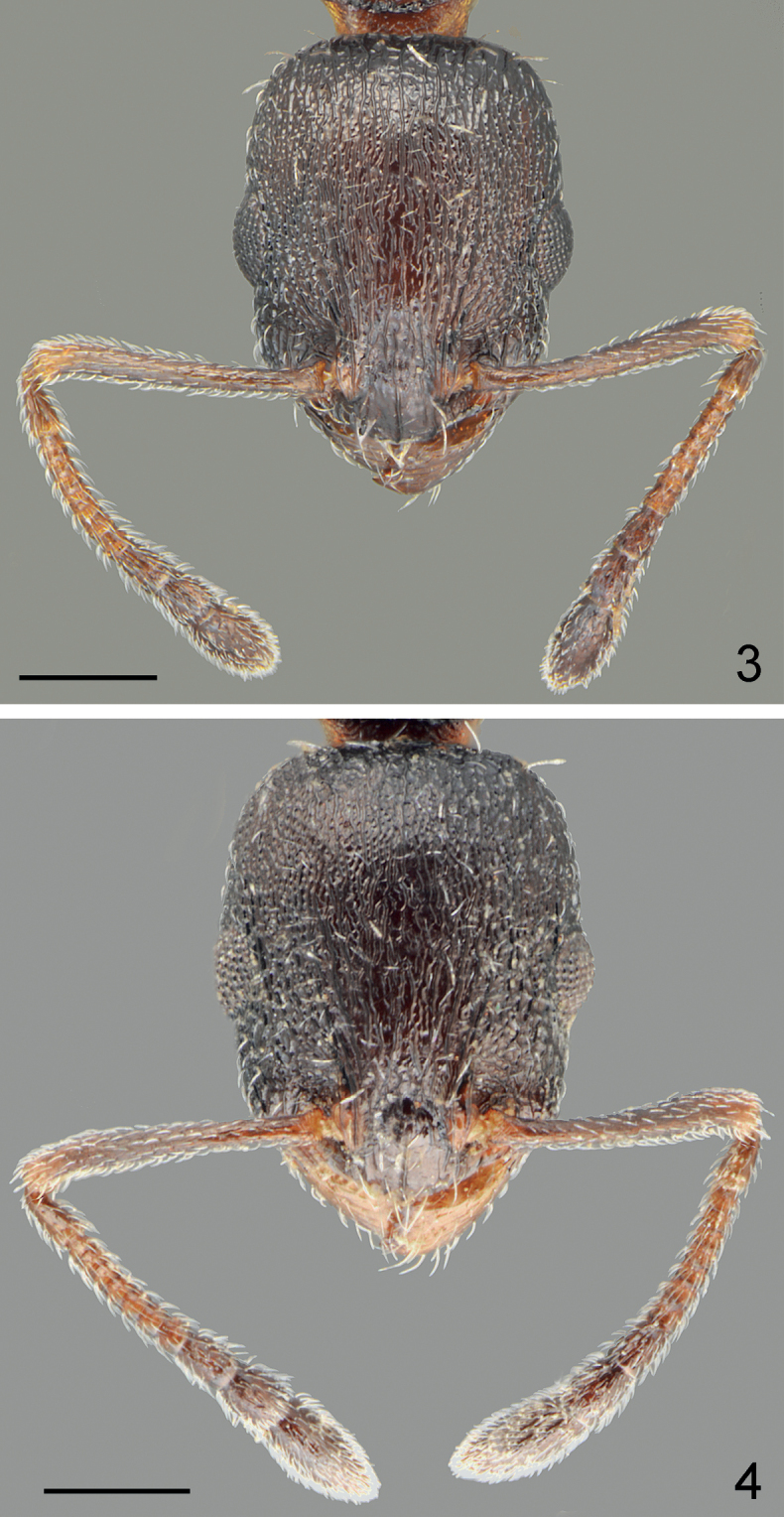
Head and antennae of holotype workers **3***Temnothoraxarkasi* sp. nov. **4***Temnothoraxeuboeae* sp. nov. Scale bars: 0.25 mm.

Figs 1–2 , 3 , 7 , 19

##### Type material.

***Holotype***: worker (CASENT4015000, pin), label: “Greece, Peloponnes | Prov. Arkadia | A. Schulz & K. Vock lgt. || Parnon, | 3 km W Sitena | 37°18'N, 22°36'E | 25.4.2000 1700 m || Collection L. Borowiec | Formicidae | LBC-GR02714” (MNHW-DBET). ***Paratypes***: 3 workers (CASENT4015001–CASENT4015003): the same data as holotype; 5 workers (CASENT4015004–CASENT4015008): the same data as holotype + “Sample Nr. | AS1”; 8 workers (CASENT4015009–CASENT4015016): the same data as holotype + “Sample Nr. | AS2”; 6 workers (CASENT4015017–CASENT4015022): the same data as holotype + “Sample Nr. | AS3” (MHNG, MNHW-DBET, PW).

##### Type locality.

Greece, Peloponnes Province, Parnon, 3 km W Sitena, 37.3/22.6, 1375 m a.s.l.

##### Differential diagnosis.

*Temnothoraxarkasi* differs from *T.parnonensis* and *T.anodontoides* in very dark body coloration, with head and mesosoma predominantly dark brown to black (pale brown to yellowish brown in both relatives) and more elongate head, i.e., 1.25–1.28 as long as wide (only 1.22 in both relatives); from *T.anodontoides* it differs additionally in more sculptured head with rugulocostulate frontal part of head (*T.anodontoides* has frons entirely rugulate); from *T.parnonensis* it additionally differs in reduced propodeal spines and lack of smooth patch on frons (*T.parnonensis* has small but well-marked triangular propodeal spines and its frons sculpture is reduced on the central part); from *T.euboeae* it differs in almost reduced propodeal spines that are in form of small angulation of very short triangular spines, shiny interspaces between head sculpture, and longer petiole with moderately elongate pedicel (*T.euboeae* has propodeal spines distinct and in form of small, short, needles, more dull head surface and very short pedicel); from *T.ikarosi* it differs in more elongate head, shorter petiolar peduncle, denser and thinner sculpture on promesonotal dorsum and smaller propodeal spines (*T.ikarosi* has less elongate head, longer petiolar peduncle, sparser and thicker promesonotal sculpture and bigger propodeal spines).

##### Description.

Worker (*N* = 10): HL: 0.66 ± 0.03 (0.6–0.71); HW: 0.55 ± 0.03 (0.5–0.57); SL: 0.49 ± 0.03 (0.44–0.53); EL: 0.14 ± 0.01 (0.11–0.16); EW: 0.1 ± 0.01 (0.08–0.12); WL: 0.8 ± 0.06 (0.68–0.89); PSL: 0.12 ± 0.01 (0.11–0.13); SDL: 0.11 ± 0.01 (0.1–0.12); PEL: 0.29 ± 0.03 (0.24–0.34); PPL: 0.17 ± 0.01 (0.15–0.19); PEH: 0.2 ± 0.02 (0.17–0.23); PPH: 0.19 ± 0.01 (0.17–0.21); PNW: 0.39 ± 0.02 (0.36–0.42); PEW: 0.18 ± 0.01 (0.15–0.19); PPW: 0.22 ± 0.02 (0.2–0.24); CI: 1.2 ± 0.03 (1.18–1.25); SI1: 0.74 ± 0.03 (0.68–0.77); SI2: 0.9 ± 0.03 (0.82–0.94); MI: 0.49 ± 0.03 (0.45–0.54); EI1: 0.75 ± 0.06 (0.67–0.85); EI2: 0.16 ± 0.01 (0.13–0.17); PI: 1.43 ± 0.1 (1.26–1.59); PPI: 0.87 ± 0.04 (0.8–0.94); PSI: 1.1 ± 0.03 (1.08–1.18).

**Colour.** Head dark brown, mesosoma, petiole and postpetiole brown to brownish black, lateral sides of pronotum with indistinct brownish areas, gaster mostly brown only base of first segment slightly brighter, in the palest specimens mesosoma partly yellowish brown; scape yellowish to yellowish brown, funicle segments 1–8 yellowish, antennal club yellowish brown to dark brown, legs mostly yellowish to yellowish brown, femora medially darkened (Figs 1, 2). ***Head*.** Slightly elongate, 1.28 times as long as wide, sides below and above eyes gently convex, occipital corners regularly rounded, occipital margin of head straight (Figs 3, 7). Anterior margin of clypeus distinctly convex, medial notch absent. Eyes moderate, short oval, 1.2 times as long as wide. Antennal scape short, in lateral view slightly curved, 0.78 times as long as length of the head, in apex gradually widened, its base without tooth, funiculus long, club 3-segmented (Fig. 3). Surface of scape finely microreticulate, shiny, covered with thin, dense, decumbent to suberect setae. Funicle longer than scape, first segment 2.1 times as long as wide at apex, segments 2–7 short, rectangular. Mandibles rounded with thick and sparse striae, shiny. Clypeus with sharp median longitudinal keel and two keels laterally, area between keels microreticulate but shiny. Frontal carinae short, slightly extending beyond frontal lobes. Antennal fossa deep, with thin circular striae and dense microreticulation. Frontal lobes narrow, microreticulate with costulae (Fig. 7). Frons, gena, malar region, vertex and temples densely reticulate with shiny interspaces; frons, gena, area behind eyes, central part of vertex, occipital area with additional costulae, malar area with costulae partly interrupted. Whole surface of head appears shiny. Sides of head with very short and sparse adpressed pubescence, sides of frons, vertex and occipital area with erect, pale, short and thick setae (Figs 3, 7). ***Mesosoma*.** Elongate, approximately twice as long as wide, distinctly arched in profile. Metanotal groove absent. Pronotum convex on sides. Propodeal spines very short, in form of triangular denticles or small angulation (Fig. 2). Whole surface of mesosoma densely reticulate with shiny interspaces. Pronotal dorsum regulate, lateral sides of pronotum rugocostulate, mesonotal dorsum reticulocostulate, propodeum rugocostulate, area below spines microreticulate with few transverse costulae. Entire mesosoma with erect, pale, moderately long and thick setae (Figs 1, 2). ***Petiole*.** In lateral view, with moderately elongate peduncle, node low, with anterior face shallowly concave and dorsum regularly rounded, whole surface rugoreticulate. Dorsal surface with sparse, short, erect setae. ***Postpetiole*.** In lateral view regularly convex, sides rounded, on the whole surface reticulocostulate, surface appears less rugose than surface of petiole. Dorsal surface with sparse, moderately long, erect setae (Figs 1, 2). ***Gaster*.** Smooth and shiny, with erect, thin, pale setae (Figs 1, 2). ***Legs*.** Moderately elongate, femora swollen in the middle, tibiae widened from base to ¾ length, surface of legs covered with sparse, adpressed to decumbent hairs.

##### Etymology.

The name is a noun in genitive case, dedicated to Arkás (ancient Greek: Ἀρκάς), a mythological hunter and King of ancient Arkadía (ancient Greek: Ἀρκαδία). His name was given to the recent Greek province Arcadia, Peloponnese, a terra typica for *Temnothoraxarkasi*.

##### Biology.

The type locality is placed in an alpine zone on the rocky northern slopes of Mt. Parnon overgrown with a young and sparse fir forest. The altitude indicated on the labels (1700 m) is most likely overestimated as the site indicated by the geographical coordinates given on the label gives the actual altitude of 1375 m.

#### ﻿ Temnothorax euboeae sp. nov.

Taxon classificationAnimaliaHymenopteraFormicidae

8A531713-A34E-559A-8C43-FDB02C45AC44

http://zoobank.org/7BF704E0-F549-4B81-8640-9FDE9F4FBDF6

**Figures 5, 6.**
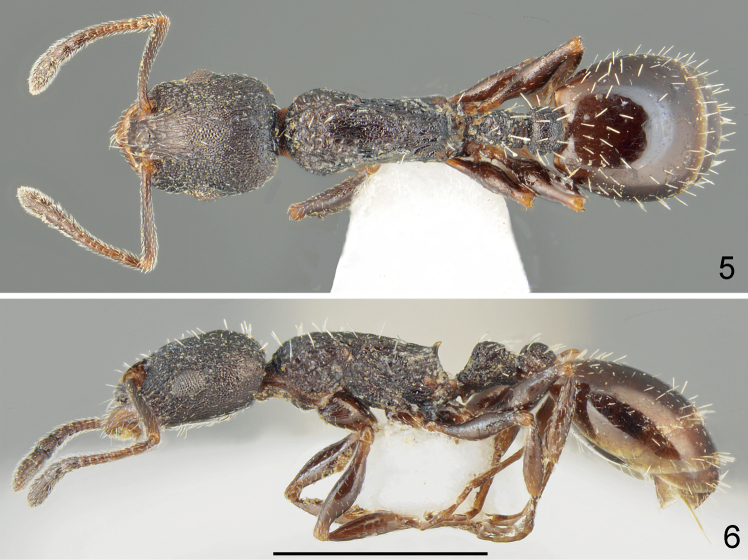
Holotype worker of *Temnothoraxeuboeae* sp. nov. **5** dorsal **6** lateral. Scale bar: 1 mm.

Figs 4 , 5–6 , 8 , 19

##### Type material.

***Holotype***: worker (CASENT4015023, pin), label: “GREECE, Sterea Ellas | Euboea, Mt. Dirfi, 1030 m | 14 V 2017 | C. Lebas || Collection L. Borowiec | Formicidae | LBC-GR02765” (MNHW-DBET).

##### Type locality.

Greece, Sterea Ellas Province, Euboea, Mt. Dirfi, 38.61666/23.83333, 1030 m a.s.l.

##### Differential diagnosis.

*Temnothoraxeuboeae* differs from *T.parnonensis* and *T.anodontoides* in very dark body coloration, with head and mesosoma predominantly dark brown to black (pale brown to yellowish brown in both relatives), more elongated head (1.25–1.28 times as long as wide vs 1.22 in both relatives), and costate frons with microreticulate interspaces (interspecies smooth in both relatives); from *T.saxatilis* it differs in very dark body coloration, with head and mesosoma predominantly dark brown to black, more coarse sculpture of mesosoma, petiole and postpetiole, and costate frons with microreticulate interspaces; from *T.anodontoides* it differs additionally in presence of propodeal spines; from *T.parnonensis* it differs additionally in shorter petiolar node; from *T.arkasi* it differs in presence of small, short, and needle shaped propodeal spines (in *T.arkasi* propodeal spines are in form of small angulation or very short triangular spines), and shorter petiole and pedicel; from *T.ikarosi* it differs in more elongated head, short petiolar peduncle, more rounded and sculptured petiole and postpetiole, lobes and short, needle shaped propodeal spines.

**Figures 7, 8.**
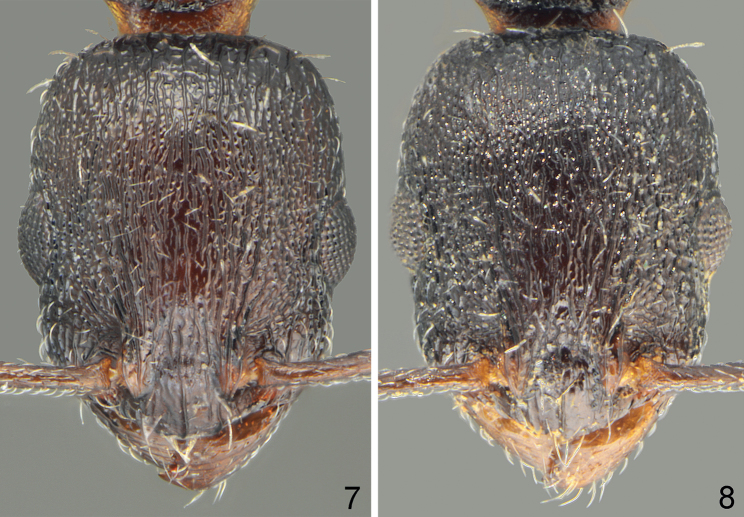
Head sculpture of holotype workers **7***Temnothoraxarkasi* sp. nov. **8***Temnothoraxeuboeae* sp. nov.

##### Description.

Worker (*N* = 1): HL: 0.7; HW: 0.57; SL: 0.44; EL: 0.14; EW: 0.09; WL: 0.87; PSL: 0.15; SDL: 0.13; PEL: 0.3; PPL: 0.17; PEH: 0.2; PPH: 0.2; PNW: 0.41; PW: 0.17; PPW: 0.25; CI: 1.23; SI1: 0.63; SI2: 0.77; MI: 0.47; EI1: 0.64; EI2: 0.13; PI: 1.5; PPI: 0.85; PSI: 1.15.

**Colour.** Head, mesosoma, petiole and postpetiole black, sides of pronotum with indistinct brownish-black areas, gaster mostly dark brown only base of first segment slightly brighter, scape brown, funicle segments 1–8 yellowish brown, antennal club dark brown, legs mostly dark brown with yellowish-brown coxae and knee, and yellowish-brown tarsi (Figs 5, 6). ***Head*.** Slightly elongate, 1.25 times as long as wide, sides below and above eyes gently convex, occipital corners regularly rounded, occipital margin of head straight (Figs 4, 8). Anterior margin of clypeus distinctly convex, medial notch absent. Eyes moderate, short oval, 1.2 times as long as wide. Antennal scape short, in lateral view slightly curved, 0.74 times as long as length of the head, in apex gradually widened, its base without tooth, funiculus long, club 3-segmented (Fig. 4). Surface of scape finely microreticulate, shiny, covered with thin, dense, decumbent to suberect setae. Funicle longer than scape, first segment 2.2 times as long as wide at apex, segments 2–7 short, rectangular. Mandibles rounded with thick and sparse striae, shiny. Clypeus with sharp median longitudinal keel and two keels laterally, area between keels microreticulate but shiny. Frontal carinae short, slightly extending beyond frontal lobes. Antennal fossa deep, with thin circular striae and dense microreticulation. Frontal lobes narrow, microreticulate with costulae (Fig. 8). Frons, gena, malar region, vertex and temples densely reticulate with dull interspaces; frons and vertex medially, gena, and area behind eyes with additional costulae, sides of frons and malar area with additional interrupted costulae, on vertex costulae fading but reticulation tends to be more longitudinal, occipital area partly with additional costulae. Whole surface of head appears slightly dull. Sides of head with very short and sparse adpressed pubescence, sides of frons, vertex and occipital area with erect, pale, short and thick setae (Figs 4, 8). ***Mesosoma*.** Elongated, approximately twice as long as wide, slightly arched in profile. Metanotal groove absent. Pronotum convex on sides. Propodeal spines short, needle shaped, directed distinctly upward, base narrow, tips sharp (Fig. 6). Whole surface of mesosoma densely rugulate with shiny interspaces. Promesonotal dorsum regulate but rugocostulate on lateral sides are more longitudinal, propodeum rugocostulate and only area between spines microreticulate. Entire mesosoma with erect, pale, moderately long and thick setae (Figs 5, 6). ***Petiole*.** In lateral view, with short peduncle, low node, with anterior face shallowly concave and dorsum regularly rounded, whole surface rugoreticulate. Dorsal surface with sparse, short, erect setae. ***Postpetiole*.** In lateral view regularly convex, sides rounded, on the whole surface reticulate, on sides with short costulae, surface appears less rugose than surface of petiole. Dorsal surface with sparse, moderately long, erect setae (Figs 5, 6). ***Gaster*.** Smooth and shiny, with erect, thin, pale setae (Figs 5, 6). ***Legs*.** Moderately elongate, femora swollen in the middle, tibiae widened from base to ¾ length, surface of legs covered with sparse, adpressed to decumbent hairs.

##### Etymology.

The name is a noun in genitive case, dedicated to Euboea, a mythical Naiad nymph whose name was given to the island of Euboea, terra typica for *Temnothoraxeuboeae*.

##### Biology.

Little known. The type locality is located in a mountainous area of Mt. Dirfi overgrown with Mediterranean oak forest.

##### Note.

We decided to describe this species based on a single specimen because of a compilation of morphological characters that make it unique among all known Greek and eastern Mediterranean *Temnothorax* species. A morphologically similar species outside eastern Mediterranean is *T.saxatilis*, known from the alpine zone in the L’Aquila province in Italy. However, *T.euboeae* differs morphologically from *T.saxatilis* based on the set of characters mentioned above in the differential diagnosis.

#### ﻿ Temnothorax parnonensis sp. nov.

Taxon classificationAnimaliaHymenopteraFormicidae

5996FA0A-CD2A-5C0A-A33E-46B1C7B04C20

http://zoobank.org/A2C4112E-A11F-4247-9325-3B0AB7BF477F

**Figures 9, 10.**
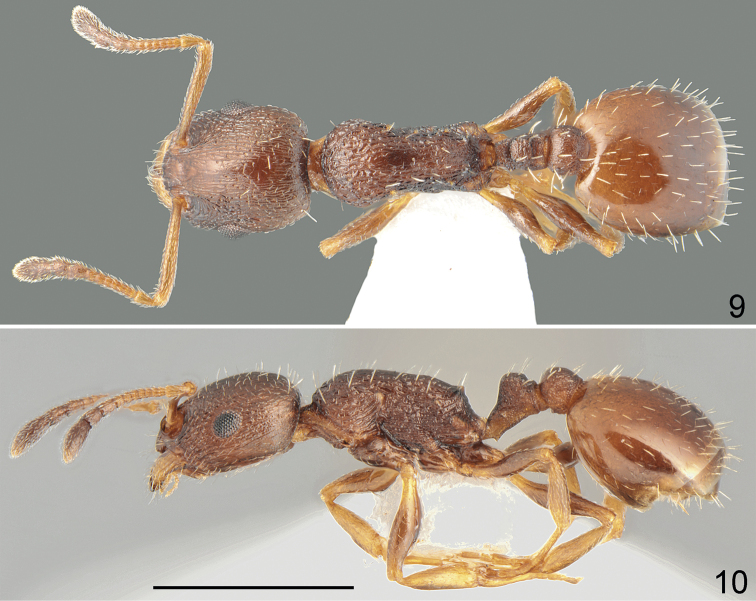
Holotype worker of *Temnothoraxparnonensis* sp. nov. **9** dorsal **10** lateral. Scale bar: 1 mm.

**Figures 11, 12.**
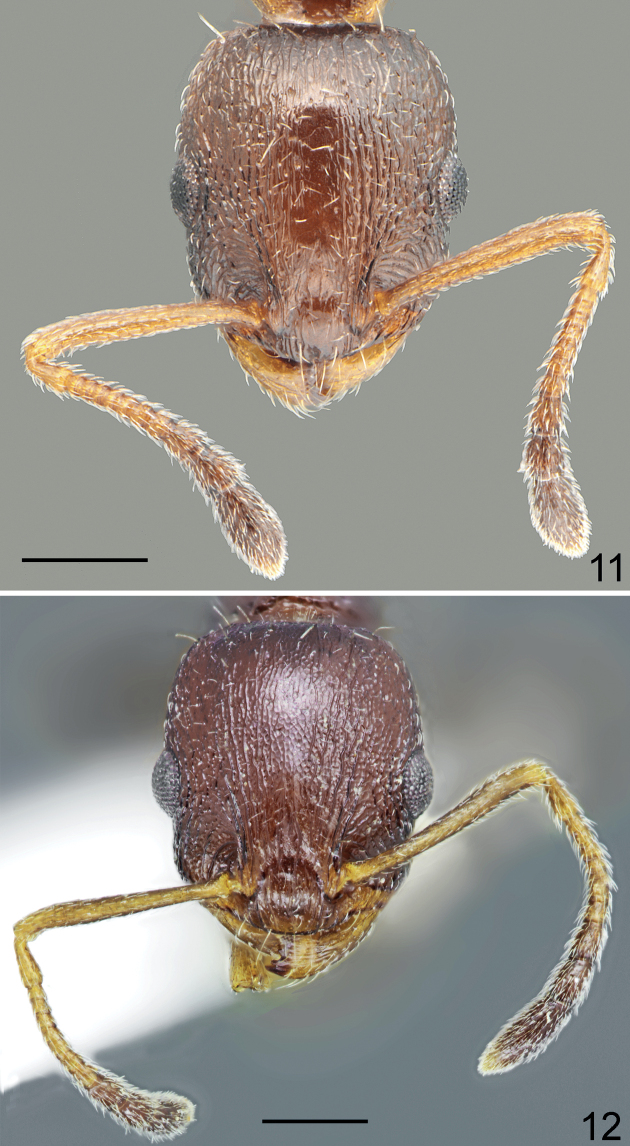
Head and antennae of workers **11***Temnothoraxparnonensis* sp. nov., holotype **12***Temnothoraxanodontoides* Dlussky & Zabelin, 1985, paratype. Scale bars: 0.25 mm.

Figs 9–10 , 11 , 15 , 19

##### Type material.

***Holotype***: worker (CASENT4015024, pin), label: “Greece, Peloponnes | Prov. Arkadia | A. Schulz & K. Vock lgt. || Parnon, | 4 km WSW Kastanitsa | 37°17'N, 22°40'E | 22.4.2000 1200–1400 m || Collection L. Borowiec | Formicidae | LBC-GR02712” (MNHW-DBET). ***Paratypes***: 3 workers (CASENT4015025–CASENT4015027): the same data as holotype; 6 workers (CASENT4015028–CASENT4015033): the same data as holotype + “Sample Nr. | AS7”; 5 paratype workers (CASENT4015034–CASENT4015038): “Greece, Peloponnes | Prov. Arkadia | A. Schulz & K. Vock lgt. || Parnon, | 3 km W Sitena| 37°18'N, 22°36'E | 25.4.2000 1700 m || Sample Nr. | AS8”; 2 workers (CASENT4015039–CASENT4015040): “Greece, Peloponnes | Prov. Arkadia | A. Schulz & K. Vock lgt. || Oros Melanon, |10 km S Levidi| 37°38'N, 22°17'E | 27.4.2000 1700 m || Collection L. Borowiec | Formicidae | LBC-GR02713”; 6 workers (CASENT4015041–CASENT4015046): the same data except LBC label but + “Sample Nr. | AS5”; 6 workers (CASENT4015047–CASENT4015052): the same data except LBC label but + “Sample Nr. | AS6”; 5 workers (CASENT4015053–CASENT4015057): “Greece, Peloponnes | Prov. Lakonia | A. Schulz & K. Vock lgt. || Oros Taigetos, | 20 km SW Sparti| 36°58'N, 22°21'E | 29.4.2000 1800–2100 m || Sample Nr. | AS4” (MHNG, MNHW-DBET, PW).

##### Type locality.

Greece, Peloponnes Province: Arcadia, Parnon, 4 km WSW Kastanitsa, 37.28333 /22.66666, 550-600 m a.s.l (please see note below for altitude estimations).

##### Differential diagnosis.

*Temnothoraxparnonensis* well differs from other species of the *T.anodontoides* group in the reduced head sculpture, with presence of smooth or indistinctly microreticulate patch on the central part of frons, and brighter yellowish brown to brown body coloration (remaining members of the group have frons entirely sculptured and darker body coloration).

##### Description.

Worker (*N* = 20): HL: 0.67 ± 0.04 (0.59–0.75); HW: 0.57 ± 0.04 (0.48–0.66); SL: 0.5 ± 0.04 (0.4–0.58); EL: 0.15 ± 0.02 (0.12–0.18); EW: 0.11 ± 0.01 (0.08–0.13); WL: 0.79 ± 0.07 (0.65–0.92); PSL: 0.12 ± 0.01 (0.09–0.15); SDL: 0.1 ± 0.01 (0.08–0.12); PEL: 0.3 ± 0.03 (0.25–0.37); PPL: 0.18 ± 0.02 (0.15–0.2); PEH: 0.22 ± 0.02 (0.19–0.26); PPH: 0.2 ± 0.02 (0.17–0.24); PNW: 0.41 ± 0.03 (0.33–0.46); PEW: 0.18 ± 0.02 (0.13–0.24); PPW: 0.24 ± 0.02 (0.2–0.28); CI: 1.17 ± 0.03 (1.11–1.23); SI1: 0.74 ± 0.03 (0.68–0.78); SI2: 0.86 ± 0.03 (0.81–0.93); MI: 0.52 ± 0.02 (0.5–0.56); EI1: 0.72 ± 0.06 (0.62–0.86); EI2: 0.16 ± 0.01 (0.14–0.18); PI: 1.37 ± 0.05 (1.27–1.48); PPI: 0.89 ± 0.08 (0.75–1.06); PSI: 1.19 ± 0.14 (1.08–1.67).

**Colour.** Head, mesosoma, petiole and postpetiole yellowish brown to brown, head usually slightly darker than mesosoma, gaster mostly yellowish brown only base of first segment slightly brighter, yellowish to rusty yellow; scape and funicle segments 1–8 yellow, antennal club darkened, yellowish brown to dark, legs mostly yellowish, femora medially darkened, yellowish brown (Figs 9, 10). ***Head*.** Slightly elongate, 1.22 times as long as wide, sides below and above eyes gently convex, occipital corners regularly rounded, occipital margin of head straight (Figs 11, 15). Anterior margin of clypeus distinctly convex, medial notch absent. Eyes moderate, short oval, 1.3 times as long as wide. Antennal scape short, in lateral view slightly curved, 0.69–0.72 times as long as length of the head, in apex gradually widened, its base without tooth, funiculus long, club 3-segmented (Fig. 11). Surface of scape microreticulate, shiny, covered with thin, dense, decumbent to suberect setae. Funicle longer than scape, first segment 2.2 times as long as wide at apex, segments 2–7 short, rectangular. Mandibles rounded with thick and sparse striae, shiny. Clypeus without or with rudiment of median keel but with two keels laterally, area between keels smooth and shiny. Frontal carinae short, slightly extending beyond frontal lobes. Antennal fossa deep, with thin circular striae and diffused microreticulation. Frontal lobes narrow, indistinctly microreticulate with costulae (Fig. 11). Frons, gena, malar region, vertex and temples reticulate with shiny interspaces, frons medially smooth or with diffused sculpture, sometimes smooth patch reduced to a shiny line, rest of frons costulate; gena costulate, malar area rugulate, area behind eyes costulate, central part of vertex with less distinct rugulosocostulae, occipital area partially costulate. Whole surface of head appears shiny. Sides of head with very short and sparse adpressed pubescence, sides of frons, vertex and occipital area with erect, pale, short and thick setae (Figs 10, 11). ***Mesosoma*.** Elongate, approximately twice as long as wide, slightly arched in profile. Metanotal groove absent. Pronotum convex on sides. Propodeal spines very short, in form of triangular spines with sharp tip (Fig. 10). Whole surface of mesosoma densely reticulate with shiny interspaces, sometimes in the middle of mesonotum sculpture diffused and microreticulate. Pronotal dorsum rugulate, lateral sides of pronotum rugocostulate; mesonotal dorsum rugoreticulate, lateral sides rugocostulate; propodeum rugocostulate, area below spines rugomicroreticulate. Entire mesosoma with erect, pale, moderately long and thick setae (Figs 9, 10). ***Petiole*.** In lateral view, with moderately long peduncle, node low, regularly rounded, with anterior face distinctly concave, whole surface rugoreticulate. Dorsal surface with sparse, short, erect setae. ***Postpetiole*.** In lateral view regularly convex, sides rounded, on the whole surface rugoreticulate, surface appears less rugose than surface of petiole. Dorsal surface with sparse, moderately long, erect setae (Figs 1, 2). ***Gaster*.** Smooth and shiny, with erect, thin, pale setae (Figs 9, 10). ***Legs*.** Legs moderately elongate, femora swollen in the middle, tibiae widened from base to ¾ length, surface of legs covered with sparse, adpressed to decumbent hairs.

##### Etymology.

The species name *parnonensis* is masculine and is a Latin singular adjective in the nominative case. The name refers to Parnon Massif, a terra typica for *T.parnonensis*.

##### Biology.

Most of the collecting sites are located in mountainous areas overgrown by Mediterranean oak forest (the eastern slopes of Mt. Parnon), and young and sparse fir forest (rocky northern slopes of Mt. Parnon and Mt. Menalon). The site in Taygetos Mts is located in alpine area above the upper border of the forest zone.

##### Note.

Based on the geographical coordinates given on the labels, latitudes for some of the collecting sites were overestimated. It applies to (label data vs altitude based on coordinates): Mt. Parnon (1200–1400 m vs 550–600 m), Mt Parnos (1700 m vs 1375 m), and Mt Menalon (1700 m vs 1450 m).

#### ﻿ Temnothorax anodontoides

Taxon classificationAnimaliaHymenopteraFormicidae

(Dlussky & Zabelin, 1985)

6F7849AC-C491-563F-A897-68BD7B107D1D

Figs 13 , 14 , 16

Leptothorax anodontoides Dlussky & Zabelin, 1985: 227, fig. 5 (w.)

##### Type material.

***Paratype*** (ANTWEB1008959). C. Зaбeлин | Koпeт-Дaг | Kapa-cy, 6.V.81 || 81-171 || Paratypus Leptoth. | anodontoides | Dlussky et Zabelin.

##### Differential diagnosis.

*Temnothoraxanodontoides* is the only member of the group with entirely absent propodeal spines. Additionally, it differs from *T.arkasi*, *T.euboeae*, and *T.ikarosi* in strongly reduced sculpture on frons, and from *T.parnonensis* in lack of smooth notch on central frons.

##### Description.

Dlussky and Zabelin (1985): 227.

##### Distribution.

Kopet Dag, Turkmenistan.

##### Comments.

Despite literature records noting *T.anodontoides* from Greece (Schulz et al. 2007), we consider its presence in this country as doubtful. By courtesy of Petr Werner (Prague), we had an opportunity to study specimens collected from the site mentioned by Schulz et al. (2007) and compare them with a paratype of *T.anodontoides* and our Greek samples of members of the *anodontoides* species-group. As a result, we concluded that the samples mentioned in the above-mentioned publication should be assigned to *T.arkasi*. There is also a record of *T.anodontoides* from Sheikhmosa in Iran (AntWeb.org, CFH000026). The photographs of this specimen certainly show a species belonging to the *anodontoides* species-group. However, its body coloration and presence of very small but distinct propodeal spines could indicate that it represents yet another undescribed species. In conclusion, the distribution of verified *T.anodontoides* is most likely restricted to Kopet Dag mountains in Turkmenistan.

**Figures 13, 14.**
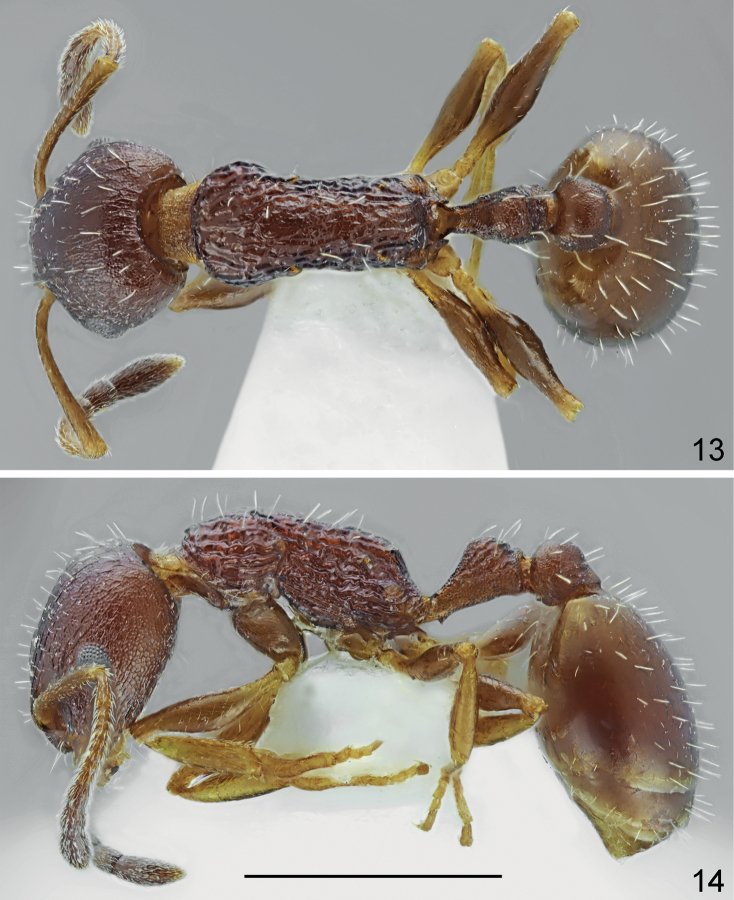
Paratype worker of *Temnothoraxanodontoides* Dlussky & Zabelin, 1985. **13** dorsal **14** lateral. Scale bar: 1 mm.

**Figures 15, 16.**
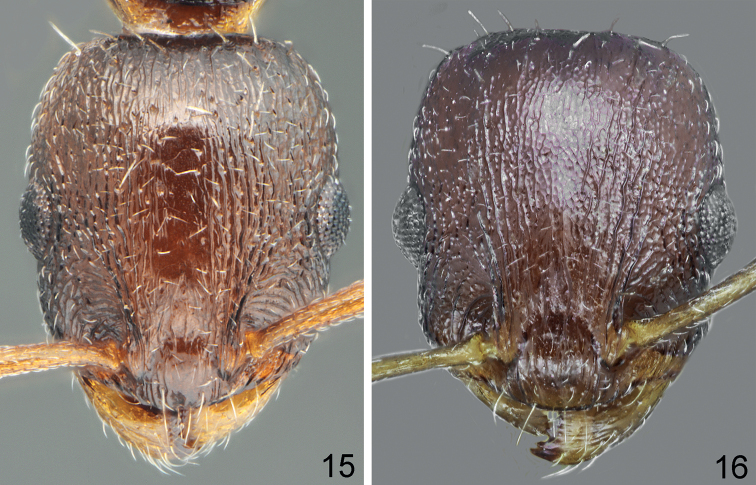
Head sculpture of workers **15***Temnothoraxparnonensis* sp. nov., holotype **16***Temnothoraxanodontoides* Dlussky & Zabelin, 1985, paratype.

#### ﻿ Temnothorax ikarosi

Taxon classificationAnimaliaHymenopteraFormicidae

Salata, Borowiec & Trichas, 2018

9DA1CD04-CBA0-5C31-A4BD-521087C44AD7

Figs 17 , 18

Temnothorax ikarosi Salata, Borowiec & Trichas, 2018: 781, figs 26–30 (w.)

##### Type material.

***Holotype*** (w.) (CASENT0845912): Temnothorax| ikarosi sp. nov. | HOLOTYPE || GREECE, Crete, Lasithi | Prov. Limnakarou Plateau | 1750 m 35°06'N, 25°28'E | 5.8.2000. M. Chatzaki (MNHW).

##### Differential diagnosis.

*Temnothoraxikarosi* differs from *T.parnonensis* in entirely sculptured head and frons lacking smooth patches; from *T.euboeae* it differs in elongated petiolar peduncle and triangular propodeal spines; from *T.anodontoides* it differs in presence of distinct propodeal spines; from *T.arkasi* it differs in less elongate head, sparser and thicker promesonotal sculpture and bigger propodeal spines.

##### Description.

Salata et al. (2018): 781.

##### Distribution.

Limnakarou Plateau, Crete, Greece.

**Figures 17, 18.**
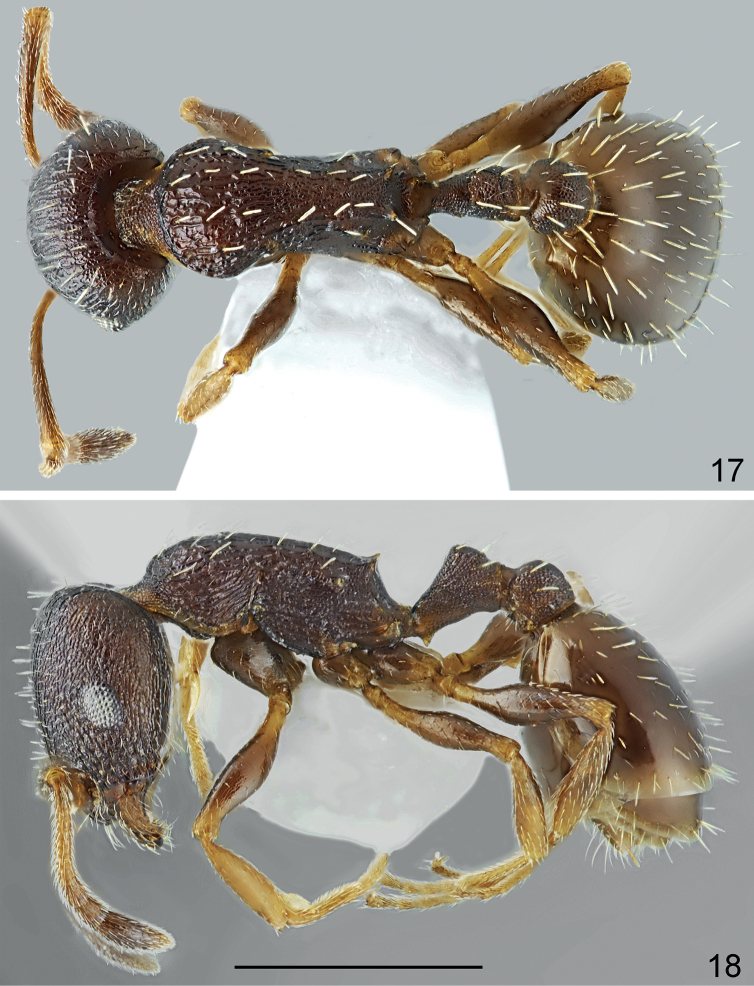
Paratype worker of *Temnothoraxikarosi* Salata, Borowiec & Trichas, 2018. **17** dorsal **18** lateral. Scale bar: 1 mm.

**Figure 19.**
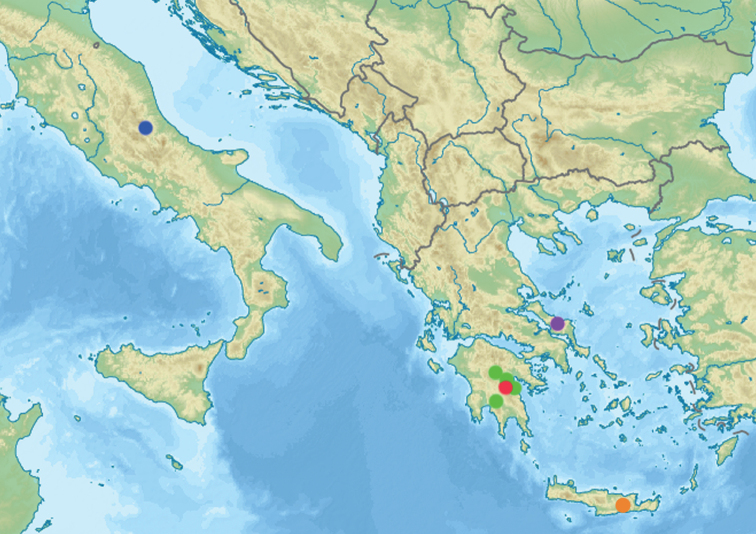
Distribution of members of the *Temnothoraxanodontoides* species-group in Europe: *Temnothoraxarkasi* (red circle), *T.euboeae* (violet circle), *T.ikarosi* (orange circle), *T.parnonensis* (green circles) and *T.saxatilis* (blue circle).

## Supplementary Material

XML Treatment for Temnothorax arkasi

XML Treatment for Temnothorax euboeae

XML Treatment for Temnothorax parnonensis

XML Treatment for Temnothorax anodontoides

XML Treatment for Temnothorax ikarosi
